# Life Course Socioeconomic Position and Cognitive Aging Trajectories: A Cross-National Cohort Study in China and England

**DOI:** 10.1093/geroni/igad064

**Published:** 2023-06-26

**Authors:** Milagros Ruiz, Yaoyue Hu, Pekka Martikainen, Martin Bobak

**Affiliations:** School of Health and Social Care, University of Essex, Colchester, UK; Research Department of Epidemiology and Public Health, University College London, London, UK; Lab 1, School of Public Health and Management, Chongqing Medical University, Chongqing, China; Population Research Unit, Faculty of Social Sciences, University of Helsinki, Helsinki, Finland; Max Planck—University of Helsinki Center for Social Inequalities in Population Health, Helsinki, Finland; Research Department of Epidemiology and Public Health, University College London, London, UK

**Keywords:** Cognitive aging, Cognitive decline, Cross-cohort study, Socioeconomic status, Socioeconomic position

## Abstract

**Background and Objectives:**

Cross-national research on cognitive aging inequality has largely concentrated on Western countries. It is unclear whether socioeconomic position (SEP) has similar effects on cognitive decline in emerging economies. We compared the association between life course SEP and cognitive function trajectories between China and England, the largest nation under state socialism and one of the oldest capitalist countries.

**Research Design and Methods:**

This cross-cohort study examined participants aged 50 years and older from the China Health and Retirement Longitudinal Study (*n* = 12,832) and the English Longitudinal Study of aging (*n* = 8,875). Cognition *z*-scores were derived using comparable measures of memory and time orientation on 4 occasions. Life course SEP was self-reported by participants at baseline. Seven- to 8-year trajectories of cognition *z*-scores were estimated using latent growth curve modeling. Country- and gender-specific associations between childhood/adolescent deprivation, education, material wealth, and home ownership were evaluated in relation to model intercept (baseline level) and linear slope (annual rate of change) of cognition.

**Results:**

After multivariable adjustment, education was positively associated with the greatest differences in baseline cognition across country and gender. Education was further linked to a slower rate of cognitive decline (*z*-score units per year); but compared with those with low education, Chinese men (*b* = 0.032) and women (*b* = 0.065) with high education had significantly slower declines than English men (*b* = −0.004) and women (*b* = 0.010) with high education.

**Discussion and Implications:**

Despite substantial between-cohort differences in downstream and upstream determinants of dementia, education provided the greatest benefits to cognitive aging in England but particularly in China.

**Translational Significance:** Given rapid worldwide population aging, a key policy aim is to target modifiable socioeconomic factors that can alleviate the burden of cognitive aging across countries with different levels of economic development and welfare systems. We compared cognitive trajectories and their association with four markers of socioeconomic position over the life course between China and England. This study found that education was most protective of cognitive function in both countries, suggesting that extending educational opportunities may promote cognitive health in older adults irrespective of the social context.

Global dementia cases are anticipated to increase nearly threefold by 2050 due to rapid population aging, thus placing a new burden on the Global South as it grapples with the social and economic impacts of dementia in the coming decades (Prince et al., 2015). This raises the question as to how ubiquitous the association between life course socioeconomic position (SEP) and cognitive aging is between different societies (Prince et al., 2015). International efforts to address dementia across the social spectrum must be guided by comparative evidence on the unequal burden and social patterning of cognitive aging and dementia in different social contexts.

Socioeconomic position (SEP) over the life course refers to different aspects of social and economic conditions that inform an individual’s place in society from early life to old age (Galobardes et al., 2006; Spiers et al., 2022). At particular life stages, SEP can also include circumstances pertaining to the family of origin and adult offspring through downward and upward intergenerational transmission (Hu & Bobak, 2018; Yang, Martikainen & Silventoinen, 2016). As a composite construct, SEP reflects measures that may influence health in context-specific ways. For instance, it has been argued that education and occupation, which are widely studied markers of SEP in high-income Western countries, are less pertinent in the Global South where socioeconomic circumstances may be better captured by material living conditions (Beckfield & Olafsdottir, 2013).

First, although individuals in disadvantaged SEPs are more likely to face worse circumstances and health in later life, one’s relative social standing is rooted in the broader structural context (Beckfield & Olafsdottir, 2013). Comparing Chinese and English adults aged ≥65, reliance on financial support from adult children and relatives is substantial in China, while there is greater dependence on assets acquired earlier in life and mature pension systems in the United Kingdom (Mason & Lee, 2018). The abundance of familial transfers could render individual wealth less important in China. Furthermore, older adults’ health and long-term care are heavily influenced by public policies (Kim, 2019). Social protection for adults aged ≥60 appears more muscular in the United Kingdom than in China, ranked 10th and 52nd, respectively, according to the 2015 Global AgeWatch Index which compared age-friendly policies between countries (Global AgeWatch Index, 2015). If greater social protection bestows more benefits to the overall population, including those most vulnerable, social inequality in cognitive aging could be less pronounced in the UK context.

Second, the cumulative effects of life course SEP on cognitive aging may be starker in some contexts. Early life deprivation lowers childhood cognitive ability, creating deficits that track into adulthood (Richards, 2014). This association partly occurs through a “chain of risk” where underprivileged children go on to have inadequate life chances as they grow up (Langenberg et al., 2006), thereby furthering their vulnerability to cognitive impairment (Turrell et al., 2002). Education boosts cognitive ability according to cognitive reserve theory (Stern, 2002) although net of this, schooling provides recognized credentials that stratify individuals across the labor market (Richards, 2014). Thus, another chain of risk ensues from lower educational attainment to poor working life and material circumstances (Langenberg et al., 2006), as disadvantaged employment narrows prospects for cognitively demanding work whereas fewer wealth curtails opportunities to engage in stimulating leisure time activities (Richards, 2014). Although each aspect of SEP has a singular influence on cognition, these effects may accumulate over the life course and widen cognitive inequality as people age. Although upward social mobility has mitigated cumulative disadvantages on cognition in some settings (Horvat et al., 2014; Turrell et al., 2002), opportunities to move up in life appear to have been more feasible in England. Although much promise has been placed on unprecedented economic development to stimulate social mobility in China and mobility trends show convergence between older adults in contemporary Chinese and British societies, substantial obstacles to upward mobility persist in China, especially for women (Li et al., 2015). Hence, trajectories of cognitive function by SEP may be wider with age in China.

A disadvantaged SEP over the life course has been associated with worse cognitive function and faster decline (Livingston et al., 2020; Richards, 2014). With growing worldwide population aging, a key scientific and policy aim is to examine the consistency of modifiable associations between socioeconomic factors and cognitive change in diverse populations. However, there is a dearth of research comparing the prospective relationship between life course SEP and cognitive aging between countries at different stages of economic development and welfare systems. To our knowledge, the present study is the first to compare trajectories in cognitive function and their association with various markers of life course SEP (childhood and adolescent deprivation, education, material wealth, and home ownership) between China and England. We add to previous longitudinal research, mainly conducted in single high-income countries, which has shown mixed associations between different markers of SEP and cognitive aging.

## Method

### Study Design

We analyzed nationally representative samples from two sister studies of aging, the China Health and Retirement Longitudinal Study (CHARLS: ≥45 years) (Zhao et al., 2014) and the English Longitudinal Study of aging (ELSA: ≥50 years) (Steptoe et al., 2013). Community-dwelling adults have been tracked in ELSA since 2002/2003 and in CHARLS since 2011. We used data measured concurrently on four occasions in CHARLS (2011, 2013, 2015, and 2018) and ELSA (2010/2011, 2014/2015, 2016/2017, and 2018/2019). Participant response rates over the study period were similar in CHARLS (2011 = 80%, 2018 = 86%; Zhao et al., 2020) and ELSA (2010/2011 = 80%, 2018/2019 = 80%; Oldfield et al., 2020). All variables were collected during the main examinations, with the exception of data on childhood which were obtained in the Life History module (CHARLS = 2014, ELSA = 2006/2007). As only a subset of the CHARLS and ELSA samples were selected to participate in the Life History interviews, we derived two analytic subsamples for each cohort. First, using data from the main examinations, we included participants aged ≥50 with data on cognition from at least one occasion for the analysis of adult SEP (CHARLS = 12,832; ELSA = 8,875) which excluded 6.1% (829/13,661) and 4.1% (382/9,257) of CHARLS and ELSA participants with no measures of cognition, respectively. Second, using the main analytic samples, we selected subsamples based on those who had completed the Life History interview for the analysis of childhood/adolescent SEP (CHARLS = 11,124; ELSA = 5,808) which excluded 13.3% (1,708/12,832) and 34.6% (3,067/8,875) of CHARLS and ELSA who were not selected for the Life History module, respectively. Statistical methods used to handle missing data on cognition (up to 3 occasions), life course SEP and baseline covariates in the analytic samples are described in a subsequent section.

### Measures

#### Cognitive function

CHARLS and ELSA had three identical cognitive tests across waves. Participants recalled as many words as possible immediately after a 10-word list was read (immediate verbal recall) and after a short delay (delayed verbal recall). The number of correctly remembered words from both trials was tallied to a score from 0 to 20. Participants also reported the: (i) day of month; (ii) month; (iii) year; and (iv) day of the week. Accurate responses were summed to a score from 0 to 4. The sum of the three cognitive tests constituted the main study outcome measure (0–24). Anterograde memory (referring to the ability to learn and retain newly encountered information) and orientation in time are fundamental cognitive functions (Kipps & Hodges, 2005) that decline with age or from more pronounced impairment caused by neurodegenerative conditions (Woodford & George, 2007).

Additional tests included in the cognitive examinations were not identical between CHARLS and ELSA. Yet, these cognitive tests encompassed other important domains, such as attention (serial sevens test = 0–5) and visuoconstruction (pentagon copy task = 0–3) in CHARLS and language in ELSA (animal naming task = 0–50; Cadar et al., 2020; Kipps & Hodges, 2005; Woodford & George, 2007). To capture the total range of cognitive functions measured across waves in each study, a supplementary outcome measure was derived by summing scores from all available domains in CHARLS (0–32) and ELSA (0–74; as per a previous CHARLS–ELSA study on cognition; Ma et al., 2020) for sensitivity analysis.

To facilitate comparability between the main and supplementary outcome measures, hereafter referred to as cognition versus total cognition, raw scores were standardized as *z*-scores. The study-specific means and standard deviations observed at the study’s baseline year (2010/2011) were used to calculate *z*-scores on four occasions for cognition (CHARLS = 10.1 ± 4.0, ELSA = 14.2 ± 4.0) and total cognition (CHARLS = 14.5 ± 5.8, ELSA = 35.0 ± 9.5). This is a widely implemented approach in longitudinal analyses of cognition *z*-scores (Ma et al., 2020; Zheng et al., 2018).

#### Life course socioeconomic position

Participants reported whether their family “ever lacked enough food to eat” in CHARLS or if they “ever experienced severe financial hardship” in ELSA, up to the age of 17 during the Life History module, with yes/no responses defining childhood/adolescent deprivation. Adult SEP was measured using data on education, material wealth, and home ownership at the present study’s baseline assessment. Data on educational attainment in CHARLS and educational qualifications in ELSA were harmonized using the 1997 International Standard Classification of Education (UNESCO Institute for Statistics, 2022): low (primary education or lower, ISCED Levels 0–1); medium (lower secondary education, ISCED Level 2); and high (upper secondary education or above, ISCED Levels 3–6). Material wealth was captured by the total number of household assets (including electronics, vehicles, and valuables), which were categorized into study-specific tertiles. Housing tenure data were dichotomized to denote whether participants (or other household members) owned their current residences.

#### Baseline covariates

Lifestyle covariates included marital/cohabitation status (*yes*/*no*); body mass index (BMI) categories (underweight, normal, overweight, and obese); smoking status (*never*, *former*, and *current*); and alcohol drinking frequency in the past year (6-point scale ranging from *never* to *almost daily or more*). Health covariates comprised the number of limitations in up to 5 activities of daily living (ADLs, Score 0–5); self-rated hearing; probable depression using thresholds for the 10-item Center for Epidemiological Depression (CES-D) scale (Andresen et al., 1994) in CHARLS (Score ≥ 12) and the 8-item CES-D scale (Turvey et al., 1999) in ELSA (Score ≥ 3); and self-reported doctor diagnosis of cardiovascular disease, hypertension, and diabetes. These covariates were selected as direct or proxy measures of risk factors which may explain an estimated 40% of the global dementia burden (Livingston et al., 2020). Although ELSA geographical data are not publicly available to researchers, all CHARLS analyses were additionally adjusted for urban–rural residence given its importance in the Chinese context.

### Statistical Analyses

#### Latent growth curve modeling

Prospective cognition trajectories (CHARLS = 7 years, ELSA = 8 years) were assessed using latent growth curve modeling (LGCM) in Mplus (version 7.4). Linear regression of the cognition *z*-scores across four occasions estimated the baseline level (intercept) and the rate of change (slope) for every year of follow-up. Longitudinal changes in cognition were slightly curvilinear in both studies, therefore, trajectories were modeled with a linear and quadratic slope.

Sequential study-specific models estimated the intercept and linear slope with the following covariates: individual SEP measure, age, and age squared (centered at 60; Model 1); Model 1 covariates plus marital status, BMI, smoking status, and drinking frequency (Model 1 + lifestyle factors); Model 1 covariates plus limitations in ADLs, hearing, probable depression, cardiovascular disease, hypertension, and diabetes (Model 1 + health factors); and all covariates (Model 2). Although the quadratic slope was statistically significant, it was not regressed on covariates due to its small effect size and little inter-individual variability. Owing to *p* values for interactions between gender with education and wealth, respectively, on the intercept (<.0001) and linear slope (education < .0001; wealth = .0195) in CHARLS, the comparative analyses were gender stratified.

#### Robustness techniques for missing data

Cognition scores were completed on four occasions for a portion of the main analytic samples (CHARLS = 43%, ELSA = 55%). Longitudinal participation patterns and associated mean scores suggested that individuals with better cognition were somewhat more likely to be followed up in both studies (Supplementary Table 1). To address attrition bias, participants with missing outcome data were retained in the analyses using full information maximum likelihood (FIML) which yields valid estimates if missingness depends on observed data (i.e., MAR) similar to multiple imputation (Graham, 2009). Repeated cognitive assessments can suffer from practice effects as individuals become more familiar with testing procedures over time, and may particularly affect younger participants (Salthouse, 2010). We predicted mean cognition scores by the number of tests completed over the follow-up in adults aged 50–59, 60–69, or ≥70, but neither study showed discernible practice effects for any age group (Supplementary Figure 1). Analytic samples included participants with missing data on SEP in childhood/adolescence (CHARLS = 2.9%, ELSA = 18.9%) and adulthood (CHARLS = 2.7%, ELSA = 5.8%). We also used FIML to deal with missingness on SEP measures and covariates by estimating their variances and covariances in stepwise models.

#### Sensitivity analysis

The main analysis is based on identical measures of anterograde memory and time orientation. To assess whether the main findings are sensitive to other cognitive domains, we replicated the main LGCMs using total cognition *z*-scores, which comprised all cognitive functions available over the study period in CHARLS (plus attention and visuoconstruction) and ELSA (plus language).

## Results

At baseline, English adults had higher memory and time orientation scores than those in China (Table 1), resulting in higher cognition scores in England compared with China. There was a consistent female disadvantage across specific cognitive domains in China. Gender differences were less clear in England; men had higher language scores, but women had higher memory scores. Although non-home ownership was slightly more prevalent in England, social disadvantage appeared more common in China with lower educational attainment and staggeringly high deprivation in childhood/adolescence.

**Table 1.** Baseline Characteristics by Country and Gender

Adult characteristics	Mean (*SD*), Median (Q1, Q3), or (%)			
	*CHARLS*—*China*		*ELSA*—*England*	
	Men (*n* = 6,362)	Women (*n* = 6,470)	Men (*n* = 3,974)	Women (*n* = 4,901)
Mean memory score (0–20)	7.0 (3.3)	6.6 (3.5)	9.9 (3.6)	10.6 (3.8)
Median time orientation score (0–4)	3 (3, 4)	3 (2, 4)	4 (4, 4)	4 (4, 4)
Mean cognition score				
Raw score (0–24)	10.1 (3.8)	9.4 (4.1)	13.7 (3.8)	14.3 (4.1)
*z*-score	0 (0.9)	−0.2 (1.0)	−0.1 (1.0)	<0.1 (1.0)
Median attention score (0–5)^a^	4.0 (1.0, 5.0)	1.0 (0, 5.0)	—	—
Median visuoconstruction score (0–3)^a^	3.0 (0, 3.0)	0 (0, 3.0)	—	—
Mean language score (0–50)^b^	—	—	20.9 (6.9)	20.5 (6.9)
Mean total cognition score				
Raw score (0–32^a^/0–74^b^)	15.6 (5.3)	13.4 (6.0)	34.7 (9.4)	34.8 (9.7)
*z*-score	0.1 (0.9)	−0.3 (1.0)	≤0.1 (1.0)	≤0.1 (1.0)
Mean age (years)	61.8 (8.0)	61.6 (8.2)	67.3 (8.8)	67.8 (9.3)
Educational level				
Low	62.4	81.1	20.7	30.7
Medium	22.6	11.5	27.0	33.5
High	15.0	7.4	52.3	35.7
Material wealth tertiles				
Low	43.6	44.9	40.8	45.6
Medium	33.1	32.6	42.3	40.0
High	23.4	22.5	16.9	14.4
Does not own current residence	9.8	11.4	14.9	17.8
Not married or cohabitating	15.8	23.7	25.3	42.2
Body mass index (kg/m^2^) categories				
Underweight	5.9	6.4	1.2	3.5
Normal	48.4	38.6	20.1	26.9
Overweight	19.4	25.3	49.7	35.8
Obese	26.3	29.7	28.9	33.8
Smoking status				
Never smoker	26.7	91.2	32.2	45.9
Past smoker	17.3	2.3	55.1	41.3
Current smoker	56.0	6.5	12.7	12.8
Alcohol drinking frequency				
Never	51.2	90.5	8.4	16.2
Less than once a month	11.3	4.9	10.8	22.1
1–3 times a month^c^/1–2 times a month^d^	5.7	1.5	10.4	12.6
1–3 times a week^c^/1–2 times a week^d^	7.2	1.1	26.2	21.3
4–6 times a week^c^/3–6 times a week^d^	1.8	0.3	23.2	14.7
Almost daily or more	22.9	1.7	21.0	13.1
Median number of limitations in activities of daily living (0–5)	0 (0, 0)	0 (0, 0)	0 (0, 0)	0 (0, 0)
Self-rated hearing				
Excellent or very good	14.1	12.7	39.5	51.4
Fair	29.5	29.4	34.1	31.9
Poor or very poor	56.5	57.9	26.4	16.7
Probable depression	23.0	36.2	18.6	26.4
Self-reported cardiovascular disease	14.6	18.5	26.8	21.3
Self-reported hypertension	27.1	31.6	43.0	40.7
Self-reported diabetes	6.1	8.1	12.8	9.1

Childhood and adolescent socioeconomic position	Men (*n* = 5,490)	Women (*n* = 5,634)	Men (*n* = 2,548)	Women (*n* = 3,260)
Ever experienced deprivation	77.8	74.0	2.8	3.1

*Notes*: CHARLS = China Health and Retirement Longitudinal Study; ELSA = English Longitudinal Study of aging; SD = standard deviation.

^a^CHARLS-specific cognition scores.

^b^ELSA-specific cognition scores.

^c^CHARLS-specific drinking frequency categories.

^d^ELSA-specific drinking frequency categories.

### Cognition Trajectories by Age

**Figure 1.**
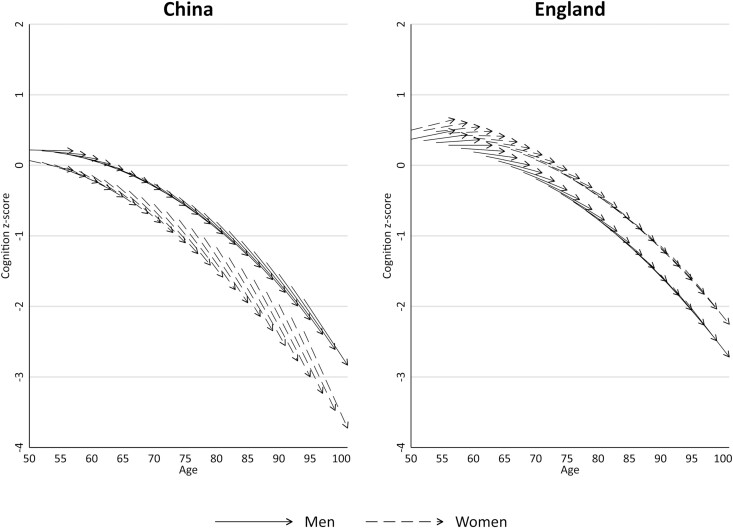
Predicted 7-year age trajectories in cognition *z*-scores according to country and gender.

Figure 1 shows 7-year trajectories in cognition for 2-year age groups at baseline (ranging from 50 to 94 years), hereafter referred to as age cohorts. Model coefficients used to estimate these trajectories are shown in Supplementary Table 2. Cognition at baseline was incrementally lower from younger to older age cohorts, and there were discernible declines in cognition during the prospective study. Nonetheless, there were several notable variations in these trends. Although baseline cognition was lower in China than in England, these country differences were much greater among women than men. Across all age cohorts, Chinese women had the lowest cognition at baseline. Both sets of trajectories indicated nonmonotonic rates of change over the follow-up, yet patterns differed between countries. Trajectories in China illustrated an acceleration in the rate of decline from younger to older age cohorts, which was underpinned by a negative linear and positive quadratic slope. By contrast, trajectories in England, expressed by a positive linear and negative quadratic slope, showed a favorable increase in cognition for cohorts aged ≤60 years before shifting to ever faster declines in older age cohorts.

### Associations With Socioeconomic Position Over the Life Course

Individuals who experienced deprivation in childhood/adolescence had lower cognition at baseline, but age-adjusted associations (Model 1) were strong only in Chinese women (Tables 2 and 3). Cognition *z*-scores were 0.037 units (95% CI: −0.084, −0.011) lower among Chinese women who reported deprivation and were partially explained by health conditions (Model 2B) rather than lifestyle factors (Model 2A). Weaker associations in England may result from inadequate power because 3% of ELSA participants reported severe financial hardship before adulthood. Although deprivation was more widespread in China, these circumstances were not associated with faster age-related cognitive declines. Cognitive decline rates per year were similarly weak by deprivation in England.

**Table 2.** Country-Specific Associations of Life Course SEP with Intercept and Linear Slope of Cognition *Z*-Scores in Men

Variable		Intercept				Linear slope			
		Model 1^a^ *SEP and age*	Model 2A^b^ *Model 1 + lifestyle*	Model 2B^c^ *Model 1 + health*	Model 3^d^ *fully adjusted*	Model 1^a^ *SEP and age*	Model 2A^b^ *Model 1 + lifestyle*	Model 2B^c^ *Model 1 + health*	Model 3^d^ *fully adjusted*
		*b* (95% CI)	*b* (95% CI)	*b* (95% CI)	*b* (95% CI)	*b* (95% CI)	*b* (95% CI)	*b* (95% CI)	*b* (95% CI)
*CHARLS*—*China*									
Childhood/ adolescent deprivation	No	Reference	Reference	Reference	Reference	Reference	Reference	Reference	Reference
	Yes	−0.037 (−0.084, 0.011)	−0.039 (−0.086, 0.008)	−0.012 (−0.059, 0.043)	−0.016 (−0.062, 0.030)	0.005 (−0.004, 0.015)	0.006 (−0.004, 0.016)	0.005 (−0.005, 0.015)	0.005 (−0.005, 0.015)
Education	Low	Reference	Reference	Reference	Reference	Reference	Reference	Reference	Reference
	Med.	0.437*** (0.394, 0.480)	0.423*** (0.379, 0.466)	0.409*** (0.366, 0.451)	0.397*** (0.355, 0.440)	0.028*** (0.019, 0.038)	0.028*** (0.018, 0.037)	0.029*** (0.020, 0.039)	0.029*** (0.019, 0.038)
	High	0.681*** (0.630, 0.732)	0.658*** (0.606, 0.709)	0.627*** (0.576, 0.678)	0.606*** (0.555, 0.657)	0.031*** (0.020, 0.043)	0.029*** (0.018, 0.041)	0.034*** (0.022, 0.045)	0.032*** (0.020, 0.043)
Material wealth	Low	Reference	Reference	Reference	Reference	Reference	Reference	Reference	Reference
	Med.	0.231*** (0.189, 0.273)	0.217*** (0.175, 0.258)	0.196*** (0.154, 0.237)	0.185*** (0.144, 0.226)	0.004 (−0.005, 0.013)	0.003 (−0.006, 0.012)	0.005 (−0.004, 0.014)	0.005 (−0.005, 0.014)
	High	0.404*** (0.356, 0.452)	0.380*** (0.332, 0.428)	0.332*** (0.284, 0.379)	0.312*** (0.264, 0.360)	0.004 (−0.007, 0.014)	0.002 (−0.008, 0.013)	0.006 (−0.004, 0.017)	0.005 (−0.005, 0.016)
Home ownership	Yes	Reference	Reference	Reference	Reference	Reference	Reference	Reference	Reference
	No	−0.011 (−0.073, 0.050)	−0.007 (−0.068, 0.054)	0.004 (−0.056, 0.064)	0.008 (−0.052, 0.068)	−0.007 (−0.021, 0.007)	−0.007 (−0.021, 0.007)	−0.008 (−0.022, 0.006)	−0.008 (−0.022, 0.006)
*ELSA*—*England*									
Childhood/ adolescent deprivation	No	Reference	Reference	Reference	Reference	Reference	Reference	Reference	Reference
	Yes	0.156 (−0.065, 0.378)	0.188 (−0.030, 0.405)	0.178 (−0.037, 0.394)	0.197 (−0.017, 0.411)	0.025 (−0.003, 0.053)	0.024 (−0.004, 0.052)	0.022 (−0.011, 0.055)	0.021 (−0.007, 0.049)
Education	Low	Reference	Reference	Reference	Reference	Reference	Reference	Reference	Reference
	Med.	0.352*** (0.228, 0.416)	0.316*** (0.251, 0.380)	0.309*** (0.246, 0.373)	0.288*** (0.225, 0.352)	−0.003 (−0.014, 0.008)	−0.005 (−0.016, 0.006)	−0.003 (−0.014, 0.007)	−0.005 (−0.016, 0.006)
	High	0.659*** (0.601, 0.717)	0.588*** (0.527, 0.648)	0.590*** (0.532, 0.648)	0.548*** (0.488, 0.608)	0.000 (−0.010, 0.010)	−0.003 (−0.013, 0.007)	−0.001 (−0.011, 0.009)	−0.004 (−0.014, 0.006)
Material wealth	Low	Reference	Reference	Reference	Reference	Reference	Reference	Reference	Reference
	Med.	0.203*** (0.152, 0.254)	0.163*** (0.112, 0.214)	0.181*** (0.130, 0.231)	0.154*** (0.104, 0.204)	−0.001 (−0.009, 0.007)	−0.001 (−0.009, 0.006)	−0.001 (−0.009, 0.007)	−0.001 (−0.009, 0.006)
	High	0.327*** (0.259, 0.394)	0.261*** (0.193, 0.328)	0.286*** (0.220, 0.352)	0.240*** (0.174, 0.307)	−0.007 (−0.017, 0.003)	−0.008 (−0.018, 0.002)	−0.007 (−0.017, 0.003)	−0.008 (−0.018, 0.002)
Home ownership	Yes	Reference	Reference	Reference	Reference	Reference	Reference	Reference	Reference
	No	−0.411*** (−0.474, −0.348)	−0.307*** (−0.375, −0.239)	−0.313*** (−0.377, −0.249)	−0.245*** (−0.313, −0.178)	−0.011 (−0.021, −0.001)	−0.007 (−0.018, −0.004)	−0.008 (−0.019, 0.002)	−0.005 (−0.016, 0.006)

*Notes*: CHARLS = China Health and Retirement Longitudinal Study; CI = confidence interval; ELSA = English Longitudinal Study of aging; SEP = socioeconomic position. The quadratic slope is not reported for brevity but is included in all models shown here. *p-value ≤ .05, **p-value ≤ .01, ***p-value ≤ .001.

^a^Intercept and linear slope were regressed on age, age squared, and individual measure of life course SEP.

^b^Intercept and linear slope were regressed on Model 1 covariates plus marital status, body mass index, smoking status, and alcohol drinking frequency.

^c^Intercept and linear slope were regressed on Model 1 covariates plus number of limitations in activities of daily living, self-rated hearing, probable depression, self-reported cardiovascular disease, hypertension, and diabetes, in turn.

^d^Intercept and linear slope were fully regressed on age, age squared, individual SEP measure, lifestyle, and health covariates.

**Table 3.** Country-Specific Associations of Life Course SEP with Intercept and Linear Slope of Cognition *Z*-Scores in Women

Variable		Intercept				Linear slope			
		Model 1^a^ *SEP and age*	Model 2A^b^ *Model 1 + lifestyle*	Model 2B^c^ *Model 1 + health*	Model 3^d^ *fully adjusted*	Model 1^a^ *SEP and age*	Model 2A^b^ *Model 1 + lifestyle*	Model 2B^c^ *Model 1 + health*	Model 3^d^ *fully adjusted*
		*b* (95% CI)	*b* (95% CI)	*b* (95% CI)	*b* (95% CI)	*b* (95% CI)	*b* (95% CI)	*b* (95% CI)	*b* (95% CI)
*CHARLS*—*China*									
Childhood/ adolescent deprivation	No	Reference	Reference	Reference	Reference	Reference	Reference	Reference	Reference
	Yes	−0.089** (−0.136, −0.041)	−0.087 (−0.134, −0.040)	−0.057 (−0.104, −0.011)	−0.056 (−0.102, −0.009)	0.000 (−0.010, 0.010)	0.000 (−0.010, 0.009)	−0.002 (−0.011, 0.008)	−0.002 (−0.012, 0.008)
Education	Low	Reference	Reference	Reference	Reference	Reference	Reference	Reference	Reference
	Med.	0.629*** (0.571, 0.686)	0.624*** (0.566, 0.682)	0.595*** (0.538, 0.652)	0.593*** (0.536, 0.650)	0.069*** (0.057, 0.082)	0.068*** (0.056, 0.080)	0.070*** (0.057, 0.082)	0.069*** (0.056, 0.081)
	High	0.978*** (0.906, 1.050)	0.978*** (0.906, 1.050)	0.924*** (0.853, 0.995)	0.925*** (0.854, 0.996)	0.067*** (0.052, 0.083)	0.065*** (0.049, 0.080)	0.068*** (0.052, 0.083)	0.065*** (0.050, 0.081)
Material wealth	Low	Reference	Reference	Reference	Reference	Reference	Reference	Reference	Reference
	Med.	0.225*** (0.181, 0.270)	0.219*** (0.174, 0.263)	0.191*** (0.147, 0.235)	0.185*** (0.142, 0.229)	0.012* (0.003, 0.022)	0.012* (0.002, 0.021)	0.013* (0.003, 0.022)	0.012* (0.003, 0.022)
	High	0.350*** (0.299, 0.401)	0.337*** (0.285, 0.389)	0.294*** (0.243, 0.345)	0.283*** (0.232, 0.334)	0.019** (0.008, 0.030)	0.016* (0.005, 0.027)	0.021** (0.010, 0.032)	0.019** (0.008, 0.030)
Home ownership	Yes	Reference	Reference	Reference	Reference	Reference	Reference	Reference	Reference
	No	−0.005 (−0.066, 0.057)	0.005 (−0.057, 0.066)	−0.001 (−0.061, 0.060)	0.007 (−0.053, 0.068)	−0.004 (−0.018, 0.010)	−0.002 (−0.016, 0.012)	−0.005 (−0.018, 0.009)	−0.003 (−0.016, 0.011)
*ELSA*—*England*									
Childhood/ adolescent deprivation	No	Reference	Reference	Reference	Reference	Reference	Reference	Reference	Reference
	Yes	0.062 (−0.101, 0.225)	0.089 (−0.070, 0.248)	0.107 (−0.053, 0.267)	0.118 (−0.039, 0.276)	−0.010 (−0.034, 0.015)	−0.009 (−0.034, 0.015)	−0.010 (−0.034, 0.015)	−0.010 (−0.034, 0.015)
Education	Low	Reference	Reference	Reference	Reference	Reference	Reference	Reference	Reference
	Med.	0.372*** (0.321, 0.424)	0.317*** (0.266, 0.369)	0.363*** (0.314, 0.412)	0.299*** (0.248, 0.351)	0.008 (0.000, 0.016)	0.006 (−0.003, 0.014)	0.008 (0.000, 0.016)	0.006 (−0.002, 0.014)
	High	0.620*** (0.568, 0.671)	0.529*** (0.475, 0.583)	0.581*** (0.526, 0.636)	0.506*** (0.452, 0.560)	0.010* (0.002, 0.018)	0.009 (0.000, 0.017)	0.011* (0.003, 0.020)	0.010 (0.001, 0.018)
Material wealth	Low	Reference	Reference	Reference	Reference	Reference	Reference	Reference	Reference
	Med.	0.210*** (0.164, 0.257)	0.151*** (0.104, 0.198)	0.166*** (0.120, 0.213)	0.129*** (0.083, 0.176)	0.002 (−0.005, 0.009)	0.001 (−0.006, 0.008)	0.002 (−0.005, 0.009)	0.001 (−0.006, 0.008)
	High	0.386*** (0.321, 0.451)	0.283*** (0.217, 0.350)	0.324*** (0.259, 0.388)	0.257*** (0.191, 0.323)	0.004 (−0.005, 0.014)	0.001 (−0.009, 0.011)	0.004 (−0.005, 0.014)	0.002 (−0.008, 0.011)
Home ownership	Yes	Reference	Reference	Reference	Reference	Reference	Reference	Reference	Reference
	No	−0.342*** (−0.397, −0.287)	−0.231*** (−0.289, −0.173)	−0.263*** (−0.318, −0.208)	−0.198*** (−0.255, −0.141)	−0.006 (−0.015, 0.003)	−0.001 (−0.010, 0.008)	−0.005 (−0.014, 0.004)	−0.001 (−0.010, 0.008)

*Notes*: CHARLS = China Health and Retirement Longitudinal Study; CI = confidence interval; ELSA = English Longitudinal Study of aging; SEP = socioeconomic position. The quadratic slope is not reported for brevity but is included in all models shown here. *p-value ≤ .05, **p-value ≤ .01, ***p-value ≤ .001.

^a^Intercept and linear slope were regressed on age, age squared, and individual measure of life course SEP.

^b^Intercept and linear slope were regressed on Model 1 covariates plus marital status, body mass index, smoking status, and alcohol drinking frequency.

^c^Intercept and linear slope were regressed on Model 1 covariates plus number of limitations in activities of daily livinga, self-rated hearing, probable depression, self-reported cardiovascular disease, hypertension, and diabetes, in turn.

^d^Intercept and linear slope were fully regressed on age, age squared, individual SEP measure, lifestyle and health covariates.

Education most strongly predicted baseline cognition across context and gender. Compared with wealth and home ownership, effect sizes by education were largest for both genders in both countries before and after adjustment. Baseline inequalities were partially explained by lifestyle and related health factors, although lifestyle played a greater role in England. Higher educational attainment was associated with slower cognitive declines in China, particularly among women. The slower rate of cognitive decline (*z*-score units per year) associated with education remained robust after full adjustment in Chinese men (medium = 0.029 95% CI: 0.019, 0.038; high = 0.032 95% CI: 0.020, 0.043) and women (medium = 0.069 95% CI: 0.056, 0.081; high = 0.065 95% CI: 0.050, 0.081). Education was less protective against cognitive aging in England as the linear decline in *z*-score units per year was more marginal, and only significantly slower for English women with high education before (0.010; 95% CI: 0.002, 0.018) and after full adjustment (0.010; 95% CI: 0.001, 0.018).

At baseline, a positive association between wealth and cognition was strong in both countries, although these gradients were shallower than those observed by education. Wealth inequalities were partially attributed to differences in lifestyle and health, but again lifestyle factors appeared more important for English adults. Despite consistent effects at baseline, wealth was not linked with better cognitive aging except for women in China. Nevertheless, the longitudinal associations with wealth in Chinese women were appreciably smaller than with education before and after full adjustment (medium = 0.012; 95% CI: 0.003, 0.022; high = 0.019; 95% CI: 0.008, 0.030).

Home ownership was associated with cognition in England, but not in China. English adults who did own their current homes had worse baseline cognition, partly due to lifestyle and health factors. Although non-home ownership was also associated with faster cognitive declines in English adults, these prospective effects were largely attenuated after further adjustment.

### Cognition Trajectories by Age and Education

**Figure 2.**
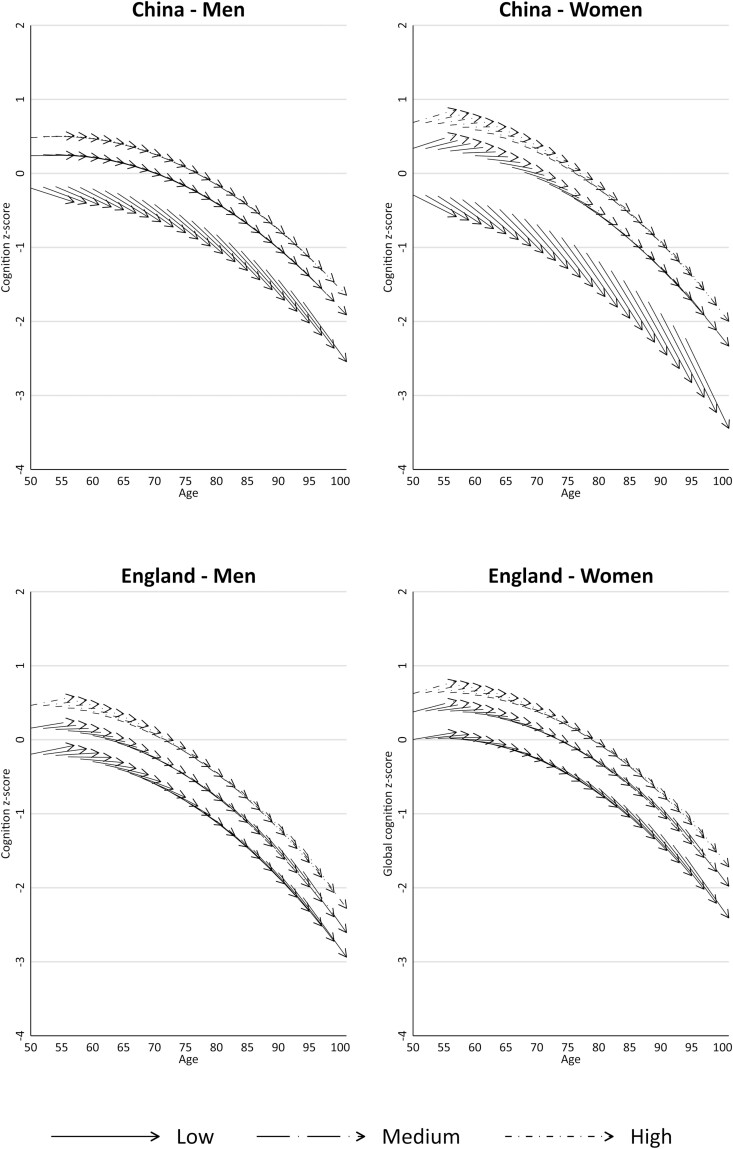
Predicted 7-year age trajectories in cognition *z*-scores by educational level according to country and gender (Model 1).

As education was the strongest predictor of cognition, Figure 2 shows 7-year trajectories for age cohorts by low, medium, and high groups using the Model 1 results. Although both countries exhibited strong educational inequalities, the cognitive health gaps between high and low groups were wider in China than in England, with the widest gaps observed for Chinese women. Trajectory patterns did not change after full adjustment (Supplementary Figure 2).

### Sensitivity Analysis

The associations between life course SEP and total cognition *z*-scores were largely similar to the main results, indicating that the differential effects of SEP are impervious to cognitive functions which were excluded from the main analysis (Supplementary Tables 3 and 4). Predicted trajectories of total cognition reiterated that education provided the greatest cognitive benefits compared with other markers of SEP (Supplementary Figures 3 and 4).

## Discussion

### Summary of Findings

Using contemporaneous data from China and England, we compared associations between life course SEP and cognitive aging trajectories. Between-country differences emerged for home ownership. Non-home-owning older adults in England had lower cognition at baseline, albeit with no effects on the subsequent decline, while in China there was no association with baseline cognition nor its decline. Between-country differences in childhood/adolescent deprivation and wealth were further modified by gender. Deprivation only hindered the baseline cognition in Chinese women. Wealth was positively associated with baseline function in all cases but only linked to slower cognitive aging in women, particularly those from China. However, cross-country similarities were overwhelmingly apparent for education, resulting in the largest inequalities in baseline cognition across the country and gender. Education further protected against cognitive decline over the 7/8-year follow-up, with stronger effects in China than in England.

### Comparison With Previous Literature

Although our current findings emphasize the importance of education on cognitive aging for older Chinese and English adults, the limited support for childhood and adolescent deprivation is perplexing because educational opportunities in young adulthood are heavily dependent on social and economic resources in the parental household. Previous investigations of path mechanisms have not looked at cognitive aging trajectories, but parental education and occupation have been shown to predict later life cognition in China and England, an association that is partly mediated by educational attainment (Richards et al., 2009; Ye et al., 2022). Our measure of childhood deprivation may perhaps not fully encapsulate early life SEP, or our findings differ due to variations in outcome measures of cognition. It is less surprising that education predicted cognitive aging more than markers of wealth given similar results from China (Wu et al., 2016) and the United States (Cagney & Lauderdale, 2002), although other evidence suggests that both markers of SEP influence baseline cognitive function (Zaninotto et al., 2018) and dementia incidence (Cadar et al., 2018) in England. Although the global body of evidence suggests that education is a leading risk factor for dementia (Livingston et al., 2020), measurement issues are more considerable for income and wealth than with education (Galobardes et al., 2006; Spiers et al., 2022), especially across social contexts (Beckfield & Olafsdottir, 2013), which adds to the difficulty in evaluating the cognitive benefits associated with material aspects of SEP.

Weaker prospective findings for deprivation, home ownership, and wealth are echoed by single studies in China and England which reported that childhood SEP, income (China), and wealth (England) were more strongly associated with baseline cognition than rates of decline (Sha et al., 2018; Yang, Martikainen, Silventoinen & Konttinen, 2016; Zaninotto et al., 2018). Contrary to our longitudinal findings for education, these trajectory studies found that educational benefits were limited to baseline levels in both countries (Yang, Martikainen, Silventoinen & Konttinen, 2016; Zaninotto et al., 2018). Incompatible findings by Yang and colleagues may reflect cohort or period effects as participants were born approximately 25 years before those in CHARLS (Yang, Martikainen, Silventoinen & Konttinen, 2016). For elderly Chinese born during the first quarter of the 20^th^ century, education provided fewer opportunities to succeed since many were already beyond prime working age during China’s economic rise. Although the ELSA-based study also tested for inequalities in cognitive decline across high, medium, and low levels of education, robust differences were only found between high and low groups and this was restricted to men (Zaninotto et al., 2018). Divergent findings may partly be due to a period effect as studies used data covering different waves (2002/2003–2010/2011 vs 2010/2011–2018/2019). The 8-year gap at baseline also makes samples less comparable. Our study included more recent generations of older adults who joined ELSA in subsequent examinations, whereas Zaninotto’s study was conducted on the original somewhat older sample. The two samples, therefore, illustrate cohort trends in educational attainment. ELSA participants with high education increased from 16% to 52% and 12% to 36%, respectively in men and women. This could have underpowered the earlier analysis because high education was treated as the reference group. Their cognition measure contained data on visuospatiality (Zaninotto et al., 2018) which was unavailable during our study period (2010/2011–2018/2019) of ELSA, which could further explain the mismatch in findings (Zaninotto et al., 2018).

The Global South has scarcely featured in cross-national prospective studies of cognitive aging inequalities. Although recent international studies, including China, reported that cross-sectional associations of cognitive function appeared stronger with education than with income (Rodriguez et al., 2021; Stefler et al., 2021), this does not clarify which aspects of SEP further protect against cognitive decline. We speculated that greater family financial transfers in China (Mason & Lee, 2018) could mitigate the negative impact of lower wealth. Indeed, non-home ownership was associated with worse baseline cognition in England but not in China, yet material wealth gradients in baseline function were similar between countries. Due to stronger social protection policies (Global AgeWatch Index, 2015; Kim, 2019) and increased avenues for upward social mobility in England (Li et al., 2015), we hypothesized that cognitive inequality would be larger in China. Net of risk factors for dementia, educational inequalities in global cognitive decline were wider in China, especially among women. China’s household registration system (“hukou”) socially differentiated persons by urban or rural origin and limited the social success of migrant workers and their families (Li et al., 2015). Older Chinese with less education were more likely to be of rural origin, and attaining the same levels of education as urbanites did not guarantee similar occupational prospects, especially for rural or migrant women (Li et al., 2015).

Although we did not set out to examine gender differences, wider educational inequalities in cognitive aging observed among older Chinese versus English women draw our attention to gender inequalities pertaining to education and other life chances. As CHARLS women had much lower levels of education than CHARLS men, as well as ELSA women, this may explain why Chinese women, particularly those with the lowest education, had the fastest declines in cognitive function. Cross-national literature on gender and health point to context-specific gendered vulnerabilities pertaining to social roles and social positions within different spheres of domestic, social, and occupational life. Female roles are often associated with certain limitations which can lead to disadvantaged social positions, although this differs from society to society (van de Velde et al., 2010). Bloomberg and colleagues suggest that the relationship between gender and education on cognitive function varies according to stages of economic development (Bloomberg et al., 2023). Our findings add to the growing literature which suggests that gender equity in education can minimize the gendered nature of cognitive decline in lower- and middle-income countries.

The salutary role of education in cognitive aging brings to mind global secular improvements in formal schooling. Although the broader literature has linked the decrease in dementia incidence across consecutive cohorts with population improvements in education, health care, nutrition and other lifestyle changes, a reversal of these cohort trends is plausible given increasing obesity, diabetes, and social isolation, coupled with declining levels of physical activity (Livingston et al., 2020). The analytic samples comprised various birth cohorts (birth year, 1910–1962 CHARLS, 1920–1959 ELSA), but the short-term follow-up precluded us from examining secular changes in education and cognitive aging and the link between these trends (Ahmadi-Abhari et al., 2017; Jagger et al., 2016; Livingston et al., 2020). Although the predicted 7-year trajectories do not distinguish age from cohort effects (i.e., age and cohort are fully collinear), a 25-year prospective study found that increasing educational attainment across successive birth cohorts in the United States was associated with better memory at baseline, but not with the subsequent decline (Dodge et al., 2017). Long-term international studies, covering different birth cohorts over the same age range, would be important to explore cohort effects in cognitive aging due to increases in the minimum school-leaving age.

Last, our findings indicated that lifestyle factors such as BMI, smoking, and drinking behaviors had less explanatory power between life course SEP and cognitive aging in China than in England. These findings illustrate East–West differences in the social patterning of health behaviors which are largely driven by cultural and social norms (Liao et al., 2019). Although targeting lifestyle factors to reduce socioeconomic inequalities in healthy cognitive aging is appropriate in some settings, healthy behavior interventions may be less effective in addressing such inequalities in China (Wang et al., 2018).

### Strengths and Limitations

Analogous data on anterograde memory and time orientation were combined to create a harmonized cognition *z*-score, which meets the assumption that being 1 *SD* below the mean in CHARLS is identical to being 1 *SD* below the mean in ELSA. Absolute cognitive levels were directly comparable between countries, thereby facilitating the comparison of within-country associations with SEP. Although the main findings may not fully reflect cognitive aging inequalities in each setting given the multidimensional nature of the cognitive function, sensitivity analysis confirmed that within-country associations with SEP did not materially change when using the total cognition *z*-score (based on all functions including those which were nonharmonizable) available in CHARLS and ELSA.

Data on adult SEP were self-reported by participants at baseline. Material wealth and home ownership in later life indicate economic resources accrued over the life course and are important markers of older people’s SEP because social advantage amasses over time (Spiers et al., 2022). Educational attainment, however, reflects early life factors such as parental SEP, is completed in young adulthood, and is predictive of future socioeconomic circumstances (Galobardes et al., 2006; Spiers et al., 2022). Data on childhood/adolescent SEP were retrospectively self-reported by participants during the Life History module, making this measure less reliable due to potential recall bias. Using a single measure of deprivation as a proxy of early life SEP is a further limitation given previous work showing the importance of parental education and occupation, among other factors (Langenberg et al., 2006; Richards, 2014). Our analysis examined each SEP marker separately, thereby ignoring the cumulative effect that life course SEP may have on cognitive aging. Unfortunately, investigating chain of risk mechanisms over the life course is not suitable using CHARLS and ELSA since data from the pre-midlife period, measured using the retrospective Life History interview, were limited to a small set of comparable health and socioeconomic measures. Finally, an important limitation of our cross-national study is that we investigated life course SEP markers which were comparable between samples. Thus, the four markers only partially capture the complexities of older adults’ socioeconomic circumstances. A broader concern is the extent to which SEP markers can feasibly reflect the lived experiences of older Chinese and English adults. Although CHARLS includes a large share of rural-dwelling adults who worked in the informal agricultural sector (Zhao et al., 2014), ELSA consists of a predominantly urban-dwelling sample employed in formal sector occupations (Steptoe et al., 2013). These differences should be considered when interpreting our findings.

Attrition bias may have underestimated cognitive inequality, but all participants with at least one cognition score over the follow-up were retained using FIML. Although a Danish study found that the association between higher education and slower cognitive decline over 10 years was not influenced by selective dropout due to death (Foverskov et al., 2018), the competing risk due to death could bias the reported associations between life course SEP and cognition trajectories in China and England. This competing risk was not incorporated in the analyses due to the lack of mortality data available over the present study period in ELSA. FIML dealt with loss to follow-up due to attrition or death under the assumption that missingness is a function of the observed analysis variables (missing at random [MAR]). Although latent growth curve models can be estimated under the assumption that missingness is related to unobserved characteristics (missing not at random [MNAR]), different modeling approaches would be required. As with MAR models, MNAR models are based on fundamentally untestable assumptions (Enders, 2011). Cognitive trajectories were curvilinear in both countries, but in England, cognition began to decline from age ≥60 and showed improvement in persons aged 50–59. This pattern supports secular improvement in cognition across cohorts in high-income countries (Tampubolon, 2016) by participants at baseline. Despite these caveats, the main strength of the present study is the comprehensive investigation of cognitive aging according to SEP across the life course in populations at different stages of economic development and welfare systems.

## Conclusion

Notwithstanding considerable differences in underlying health status and social context, chronic disease antecedents, and sociodemographic patterns of health behaviors (Horvat et al., 2014), education was beneficial for mid-to-late life cognitive resilience in China and England. Inclusive education appears an important international priority to promote healthy cognitive aging of older populations. Encompassing learning and skills acquisition from the early years to maturity, formal education can influence cognitive performance throughout the life span (Livingston et al., 2020; Richards, 2014). Long-term life course research may help to uncover windows of opportunity for educational policies in countries with growing aging populations.

## Supplementary Material

igad064_suppl_Supplementary_Material

## Data Availability

This study was made possible using the data collected by the China Health and Retirement Longitudinal Study (CHARLS) and the English Longitudinal Study of Aging (ELSA). Specifically, this research has been conducted using data from Waves 1–4 in CHARLS, and Wave 5 plus Waves 7–9 data in ELSA. These data sets are available in public, open-access repositories: (CHARLS: http://charls.pku.edu.cn/pages/data/111/en.html; ELSA: https://beta.ukdataservice.ac.uk/datacatalogue/series/series?id=200011).
