# Supplementary material for: Life Course Socioeconomic Position and Cognitive Aging Trajectories: A Cross-National Cohort Study in China and England
Source: Innov Aging. 2023 Jun 26;7(6):igad064. doi: 10.1093/geroni/igad064 (PMC10516463; doi:10.1093/geroni/igad064)

- [Supplementary Table 1 Cognition scores across participation patterns in the country-specific analytic samples 27](#_Toc107489821)
- [Supplementary Table 2 Country- and gender-specific growth model of cognition z-scores by age 28](#_Toc107489822)
- [Supplementary Table 3 Country-specific associations of life course SEP with intercept and linear slope of total cognition z-scores in men 29](#_Toc107489823)
- [Supplementary Table 4 Country-specific associations of life course SEP with intercept and linear slope of total cognition z-scores in women 29](#_Toc107489824)
- [Supplementary Figure 1 Country-specific predicted cognition z-scores by the number of cognitive tests taken stratified by age group 31](#_Toc107489825)
- [Supplementary Figure 2 Predicted 7-year age trajectories in cognition z-scores by educational level according to country and gender (Model 3) 32](#_Toc107489826)
- [Supplementary Figure 3 Predicted 7-year age trajectories in total cognition z-scores by educational level according to country and gender (Model 1) 33](#_Toc107489827)
- [Supplementary Figure 4 Predicted 7-year age trajectories in total cognition z-scores by educational level according to country and gender (Model 3) 34](#_Toc107489828)

| **N of participants (%)** | ***CHARLS-China*** | | | |
| --- | --- | --- | --- | --- |
|  | **2011** | **2013** | **2015** | **2018** |
| 5,488 (43%) | 10.3 (3.6) | 10.3 (3.9) | 9.7 (4.0) | 9.5 (4.7) |
| 1,916 (15%) | 8.9 (3.8) | 8.3 (4.1) | 7.3 (4.3) | - |
| 1,075 (8%) | 8.8 (4.4) | - | - | - |
| 809 (6%) | 9.0 (4.2) | 8.5 (4.6) | - | - |
| 798 (6%) | - | 9.2 (4.0) | 8.6 (4.0) | 8.3 (4.7) |
| 579 (5%) | 10.0 (4.1) | - | 8.7 (4.2) | 8.3 (5.0) |
| 436 (3%) | 8.4 (4.0) | - | 6.0 (4.5) | - |
| 431 (3%) | - | 7.4 (4.4) | 6.0 (4.3) | - |
| 356 (3%) | 9.8 (4.0) | 10.0 (4.2) | - | 8.5 (5.0) |
| 243 (2%) | - | 6.2 (4.6) | - | - |
| 217 (2%) | - | - | 7.5 (4.0) | 7.2 (4.7) |
| 193 (2%) | - | - | 4.3 (4.4) | - |
| 167 (1%) | 10.7 (4.4) | - | - | 9.4 (4.8) |
| 66 (<1%) | - | 8.6 (3.6) | - | 7.8 (5.1) |
| 58 (<1%) | - | - | - | 8.7 (5.0) |
| **12,832 (100%)** |  |  |  |  |
| **N of participants (%)** | ***ELSA-England*** | | | |
|  | **2011/2** | **2014/5** | **2016/7** | **2018/9** |
| 4,849 (55%) | 15.1 (3.3) | 14.8 (3.6) | 14.6 (3.7) | 14.1 (4.0) |
| 1,861 (21%) | 12.1 (4.6) | - | - | - |
| 853 (10%) | 13.5 (3.7) | 12.5 (4.2) | 11.7 (4.7) | - |
| 802 (9%) | 12.7 (4.1) | 11.2 (5.0) | - | - |
| 186 (2%) | 14.5 (3.9) | 14.1 (4.1) | - | 13.0 (4.5) |
| 136 (2%) | 14.7 (3.4) | - | 13.8 (4.1) | 13.8 (4.2) |
| 64 (<1%) | 13.0 (4.6) | - | 10.7 (5.6) | - |
| 38 (<1%) | - | 14.5 (4.3) | 14.1 (4.7) | 13.1 (4.9) |
| 25 (<1%) | 13.2 (4.8) | - | - | 11.9 (5.3) |
| 23 (<1%) | - | 9.5 (6.7) | - | - |
| 13 (<1%) | - | 10.2 (4.8) | 8.5 (4.8) | - |
| 9 (<1%) | - | - | 11.6 (3.6) | 12.7 (4.2) |
| 8 (<1%) | - | - | 9.1 (6.7) | - |
| 6 (<1%) | - | - | - | 13.5 (3.4) |
| 2 (<1%) | - | 14.5 (0.7) | - | 13.5 (3.4) |
| **8,875 (100%)** |  |  |  |  |

## Supplementary Table 1 Mean cognition scores (0-24) across participation patterns in the country-specific analytic samples

## Supplementary Table 2 Country- and gender-specific growth model of cognition z-scores by age

| **b (95% CI)** | ***CHARLS – China*** | | ***ELSA – England*** | |
| --- | --- | --- | --- | --- |
|  | **Men** | **Women** | **Men** | **Women** |
| Intercept^a^ | 0.078 (0.054, 0.101) | -0.142 (-0.167, -0.117) | 0.189 (0.159, 0.219) | 0.388 (0.360, 0.416) |
| Intercept variance | 0.373 (0.346, 0.400) | 0.465 (0.434, 0.495) | 0.416 (0.376, 0.456) | 0.451 (0.414, 0.487) |
| Intercept regressed on  Age  Age squared | -0.024 (-0.027 -0.020)  -0.001 (-0.001, 0.000) | -0.031 (-0.035, -0.028)  0.000 (-0.001, 0.000) | -0.028 (-0.033, -0.022)  -0.001 (-0.001, -0.001) | -0.021 (-0.027, -0.016)  -0.001 (-0.002, -0.001) |
| Linear slope^*^ | -0.074 (-0.085, -0.062) | -0.074 (-0.086, -0.062) | 0.002 (-0.009, 0.013) | 0.013 (0.003, 0.023) |
| Linear slope variance | 0.004 (0.002, 0.005) | 0.004 (0.003, 0.006) | 0.002 (0.001, 0.003) | 0.002 (0.001, 0.003) |
| Linear slope regressed on  Age  Age squared | -0.003 (-0.004, -0.002)  0.000 (0.000, 0.000) | -0.004 (-0.005, -0.003)  0.000 (0.000, 0.000) | -0.003 (-0.004, -0.002)  0.000 (0.000, 0.000) | -0.003 (-0.003, -0.002)  0.000 (0.000, 0.000) |
| Intercept - linear slope covariance | 0.010 (0.005, 0.015) | 0.021 (0.016, 0.026) | 0.006 (0.000, 0.011) | 0.007 (0.002, 0.012) |
| Quadratic slope | 0.006 (0.004, 0.007) | 0.002 (-0.001, 0.004) | -0.002 (-0.004, -0.001) | -0.003 (-0.005, -0.002) |
| Model fit  CFI  TLI  RMSEA | 0.982  0.968  0.044 | 0.994  0.990  0.027 | 0.996  0.993  0.026 | 0.996  0.994  0.027 |

CHARLS, China Health and Retirement Longitudinal Study; CFI, Comparative Fit Index; ELSA, English Longitudinal Study of Ageing; RMSEA, root mean-square error of approximation; TLI, Tucker–Lewis Index.

^a^Intercept and linear slope were regressed on age and age squared (both centred at 60).

## Supplementary Table 3 Country-specific associations of life course SEP with intercept and linear slope of total cognition z-scores in men

| **b (95% CI)** | | **Intercept** | | | | **Linear slope** | | | |
| --- | --- | --- | --- | --- | --- | --- | --- | --- | --- |
|  |  | **Model 1^a^:**  ***SEP and age*** | **Model 2A^b^:**  ***Model 1 + lifestyle*** | **Model 2B^c^:**  ***Model 1 + health*** | **Model 3^d^:**  ***Fully adjusted*** | **Model 1^a^:**  ***SEP and age*** | **Model 2A^b^:**  ***Model 1 + lifestyle*** | **Model 2B^c^:**  ***Model 1 + health*** | **Model 3^d^:**  ***Fully adjusted*** |
| ***CHARLS – China**** | | | | | | | | | |
| Childhood/ adolescent deprivation | No | Reference | Reference | Reference | Reference | Reference | Reference | Reference | Reference |
|  | Yes | -0.039  (-0.085, 0.008) | -0.042  (-0.088, 0.004) | -0.013  (-0.058, 0.032) | -0.019  (-0.063, 0.026) | 0.005  (-0.004, 0.014) | 0.006  (-0.003, 0.015) | 0.004  (-0.005, 0.013) | 0.005  (-0.004, 0.014) |
| Education | Low | Reference | Reference | Reference | Reference | Reference | Reference | Reference | Reference |
|  | Med. | 0.523  (0.482, 0.565) | 0.507  (0.466, 0.548) | 0.492  (0.451, 0.533) | 0.479  (0.439, 0.520) | 0.014  (0.006, 0.023) | 0.014  (0.005, 0.022) | 0.016  (0.007, 0.024) | 0.015  (0.007, 0.024) |
|  | High | 0.746  (0.697, 0.795) | 0.720  (0.671, 0.769) | 0.688  (0.640, 0.736) | 0.664  (0.616, 0.713) | 0.022  (0.012, 0.032) | 0.020  (0.005, 0.030) | 0.025  (0.015, 0.035) | 0.023  (0.013, 0.034) |
| Material wealth | Low | Reference | Reference | Reference | Reference | Reference | Reference | Reference | Reference |
|  | Med. | 0.256  (0.216, 0.297) | 0.239  (0.199, 0.280) | 0.216  (0.176, 0.255) | 0.203  (0.163, 0.242) | 0.002  (-0.006, 0.010) | 0.002  (-0.007, 0.010) | 0.004  (-0.004, 0.012) | 0.004  (-0.005, 0.012) |
|  | High | 0.451  (0.405, 0.498) | 0.424  (0.377, 0.470) | 0.371  (0.325, 0.417) | 0.348  (0.302, 0.394) | 0.001  (-0.008, 0.010) | 0.000  (-0.010, 0.009) | 0.005  (-0.004, 0.015) | 0.004  (-0.005, 0.014) |
| Home ownership | Yes | Reference | Reference | Reference | Reference | Reference | Reference | Reference | Reference |
|  | No | -0.025  (-0.084, 0.035) | -0.020  (-0.079, 0.039) | -0.007  (-0.065, 0.051) | -0.003  (-0.061, 0.055) | -0.001  (-0.014, 0.011) | -0.001  (-0.014, 0.011) | -0.002  (-0.015, 0.010) | -0.002  (-0.015, 0.010) |
| ***ELSA – England***† | | | | | | | | | |
| Childhood/ adolescent deprivation | No | Reference | Reference | Reference | Reference | Reference | Reference | Reference | Reference |
|  | Yes | 0.132  (-0.037, 0.302) | 0.119  (-0.046, 0.285) | 0.143  (-0.025, 0.310) | 0.136  (-0.028, 0.300) | 0.008  (-0.020, 0.037) | 0.007  (-0.022, 0.036) | 0.010  (-0.019, 0.039) | 0.008  (-0.020, 0.037) |
| Education | Low | Reference | Reference | Reference | Reference | Reference | Reference | Reference | Reference |
|  | Med. | 0.351  (0.285, 0.417) | 0.313  (0.247, 0.380) | 0.311  (0.245, 0.377) | 0.288  (0.222, 0.354) | 0.003  (-0.007, 0.014) | 0.002  (-0.009, 0.012) | 0.003  (-0.008, 0.013) | 0.001  (-0.009, 0.012) |
|  | High | 0.683  (0.623, 0.743) | 0.607  (0.545, 0.669) | 0.618  (0.557, 0.678) | 0.569  (0.508, 0.631) | 0.004  (-0.005, 0.014) | 0.002  (-0.008, 0.012) | 0.003  (-0.007, 0.012) | 0.001  (-0.009, 0.011) |
| Material wealth | Low | Reference | Reference | Reference | Reference | Reference | Reference | Reference | Reference |
|  | Med. | 0.193  (0.140, 0.246) | 0.149  (0.096, 0.201) | 0.170  (0.118, 0.222) | 0.139  (0.087, 0.191) | 0.003  (-0.005, 0.010) | 0.002  (-0.005, 0.010) | 0.003  (-0.005, 0.010) | 0.002  (-0.005, 0.010) |
|  | High | 0.352  (0.282, 0.421) | 0.280  (0.210, 0.349) | 0.312  (0.243, 0.380) | 0.259  (0.190, 0.328) | -0.009  (-0.019, 0.000) | -0.010  (-0.020, 0.000) | -0.010  (-0.019, 0.000) | -0.010  (-0.020, 0.000) |
| Home ownership | Yes | Reference | Reference | Reference | Reference | Reference | Reference | Reference | Reference |
|  | No | -0.397  (-0.463, -0.332) | -0.272  (-0.342, -0.201) | -0.306  (-0.372, -0.241) | -0.211  (-0.282, -0.141) | -0.012  (-0.022, -0.002) | -0.011  (-0.022, -0.001) | -0.011  (-0.025, 0.003) | -0.008  (-0.019, 0.003) |

The quadratic slope is not reported for brevity but is included in all models shown here.

*Total cognition z-scores in CHARLS are based on raw scores ranging from 0-32. Raw scores comprise memory (0-20), time orientation (0-4), numeracy (0-5), and visual construction (0-3).

†Total cognition z-scores in ELSA are based on raw scores ranging from 0-74. Raw scores comprise memory (0-20), time orientation (0-4), and language (0-50).

^a^Intercept and linear slope were regressed on age, age squared, and individual measure of life course SEP.

^b^Intercept and linear slope were regressed on Model 1 covariates plus marital status, BMI, smoking status, and alcohol drinking frequency.

^c^Intercept and linear slope were regressed on Model 1 covariates plus number of limitations in ADLs, self-rated hearing, probable depression, self-reported cardiovascular disease, hypertension, and diabetes, in turn.

^d^Intercept and linear slope were fully regressed on age, age squared, individual SEP measure, lifestyle and health covariates.

## Supplementary Table 4 Country-specific associations of life course SEP with intercept and linear slope of total cognition z-scores in women

| **b (95% CI)** | | **Intercept** | | | | **Linear slope** | | | |
| --- | --- | --- | --- | --- | --- | --- | --- | --- | --- |
|  |  | **Model 1^a^:**  ***SEP and age*** | **Model 2A^b^:**  ***Model 1 + lifestyle*** | **Model 2B^c^:**  ***Model 1 + health*** | **Model 3^d^:**  ***Fully adjusted*** | **Model 1^a^:**  ***SEP and age*** | **Model 2A^b^:**  ***Model 1 + lifestyle*** | **Model 2B^c^:**  ***Model 1 + health*** | **Model 3^d^:**  ***Fully adjusted*** |
| ***CHARLS – China**** | | | | | | | | | |
| Childhood/  adolescent deprivation | No | Reference | Reference | Reference | Reference | Reference | Reference | Reference | Reference |
|  | Yes | -0.108  (-0.156, -0.061) | -0.108  (-0.156, -0.060) | -0.074  (-0.120, -0.027) | -0.073  (-0.120, -0.027) | 0.005  (-0.004, 0.014) | 0.005  (-0.004, 0.014) | 0.004  (-0.005, 0.013) | 0.003  (-0.006, 0.012) |
| Education | Low | Reference | Reference | Reference | Reference | Reference | Reference | Reference | Reference |
|  | Med. | 0.815  (0.758, 0.872) | 0.808  (0.751, 0.864) | 0.775  (0.719, 0.831) | 0.771  (0.715, 0.826) | 0.045  (0.034, 0.056) | 0.044  (0.033, 0.055) | 0.046  (0.035, 0.057) | 0.045  (0.035, 0.056) |
|  | High | 1.127  (1.057, 1.197) | 1.123  (1.053, 1.194) | 1.063  (0.994, 1.133) | 1.062  (0.993, 1.131) | 0.043  (0.029, 0.056) | 0.041  (0.028, 0.054) | 0.044  (0.031, 0.058) | 0.043  (0.029, 0.056) |
| Material wealth | Low | Reference | Reference | Reference | Reference | Reference | Reference | Reference | Reference |
|  | Med. | 0.282  (0.238, 0.327) | 0.274  (0.230, 0.319) | 0.243  (0.199, 0.286) | 0.236  (0.192, 0.280) | 0.005  (-0.003, 0.014) | 0.005  (-0.004, 0.014) | 0.007  (-0.002, 0.015) | 0.007  (-0.002, 0.015) |
|  | High | 0.446  (0.395, 0.498) | 0.429  (0.378, 0.481) | 0.384  (0.333, 0.435) | 0.370  (0.319, 0.421) | 0.008  (-0.002, 0.017) | 0.006  (-0.004, 0.016) | 0.011  (0.001, 0.021) | 0.009  (0.000, 0.019) |
| Home ownership | Yes | Reference | Reference | Reference | Reference | Reference | Reference | Reference | Reference |
|  | No | -0.011  (-0.073, 0.051) | 0.000  (-0.061, 0.062) | -0.008  (-0.069, 0.052) | 0.002  (-0.059, 0.062) | -0.002  (-0.015, 0.010) | -0.001  (-0.014, 0.012) | -0.003  (-0.015, 0.010) | -0.001  (-0.014, 0.011) |
| ***ELSA – England***† | | | | | | | | | |
| Childhood/  adolescent deprivation | No | Reference | Reference | Reference | Reference | Reference | Reference | Reference | Reference |
|  | Yes | -0.012  (-0.157, 0.133) | 0.002  (-0.140, 0.145) | 0.003  (-0.140, 0.146) | 0.009  (-0.132, 0.150) | 0.003  (-0.021, 0.027) | 0.003  (-0.021, 0.026) | 0.004  (-0.019, 0.028) | 0.004  (-0.020, 0.027) |
| Education | Low | Reference | Reference | Reference | Reference | Reference | Reference | Reference | Reference |
|  | Med. | 0.408  (0.357, 0.459) | 0.349  (0.297, 0.401) | 0.368  (0.317, 0.418) | 0.331  (0.279, 0.382) | 0.007  (0.000, 0.015) | 0.006  (-0.002, 0.014) | 0.006  (-0.002, 0.014) | 0.005  (-0.003, 0.013) |
|  | High | 0.730  (0.678, 0.781) | 0.635  (0.581, 0.688) | 0.674  (0.622, 0.725) | 0.611  (0.558, 0.665) | 0.004  (-0.004, 0.012) | 0.002  (-0.006, 0.010) | 0.003  (-0.005, 0.011) | 0.002  (-0.006, 0.010) |
| Material wealth | Low | Reference | Reference | Reference | Reference | Reference | Reference | Reference | Reference |
|  | Med. | 0.261  (0.213, 0.308) | 0.198  (0.151, 0.245) | 0.216  (0.169, 0.262) | 0.176  (0.129, 0.223) | -0.001  (-0.008, 0.006) | -0.002  (-0.009, 0.004) | -0.002  (-0.009, 0.005) | -0.003  (-0.009, 0.004) |
|  | High | 0.409  (0.343, 0.474) | 0.298  (0.232, 0.365) | 0.344  (0.279, 0.409) | 0.271  (0.205, 0.337) | 0.004  (-0.005, 0.013) | 0.001  (-0.008, 0.011) | 0.003  (-0.006, 0.012) | 0.001  (-0.008, 0.010) |
| Home ownership | Yes | Reference | Reference | Reference | Reference | Reference | Reference | Reference | Reference |
|  | No | -0.339  (-0.394, -0.283) | -0.214  (-0.272, -0.156) | -0.266  (-0.320, -0.211) | -0.181  (-0.239, -0.124) | -0.011  (-0.020, -0.003) | -0.008  (-0.017, 0.001) | -0.010  (-0.021, 0.002) | -0.007  (-0.016, 0.002) |

The quadratic slope is not reported for brevity but is included in all models shown here.

*Total cognition z-scores in CHARLS are based on raw scores ranging from 0-32. Raw scores comprise memory (0-20), time orientation (0-4), numeracy (0-5), and visual construction (0-3).

†Total cognition z-scores in ELSA are based on raw scores ranging from 0-74. Raw scores comprise memory (0-20), time orientation (0-4), and language (0-50).

^a^Intercept and linear slope were regressed on age, age squared, and individual measure of life course SEP.

^b^Intercept and linear slope were regressed on Model 1 covariates plus marital status, BMI, smoking status, and alcohol drinking frequency.

^c^Intercept and linear slope were regressed on Model 1 covariates plus number of limitations in ADLs, self-rated hearing, probable depression, self-reported cardiovascular disease, hypertension, and diabetes, in turn.

^d^Intercept and linear slope were fully regressed on age, age squared, individual SEP measure, lifestyle and health covariates.

## Supplementary Figure 1 Country-specific predicted cognition z-scores by the number of cognitive tests taken stratified by age group


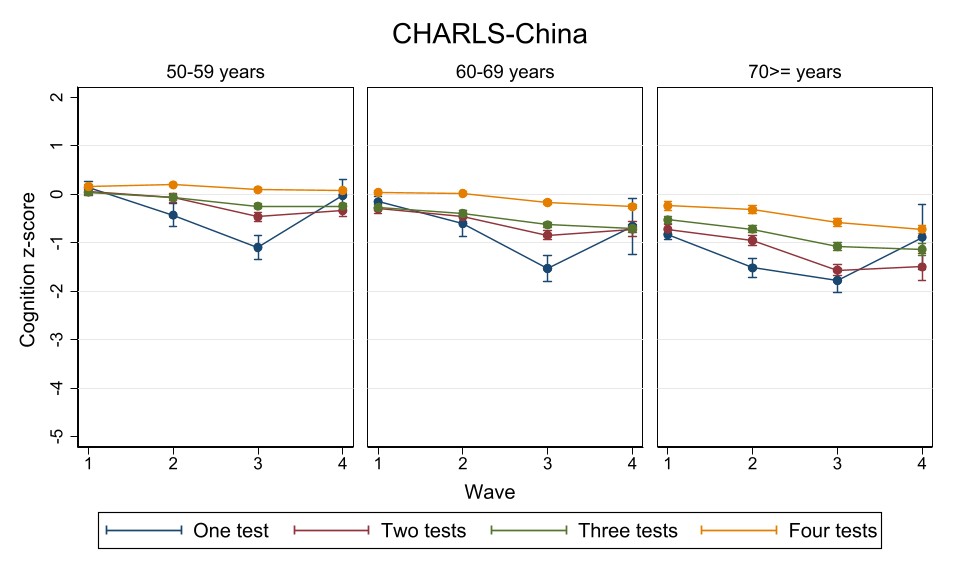


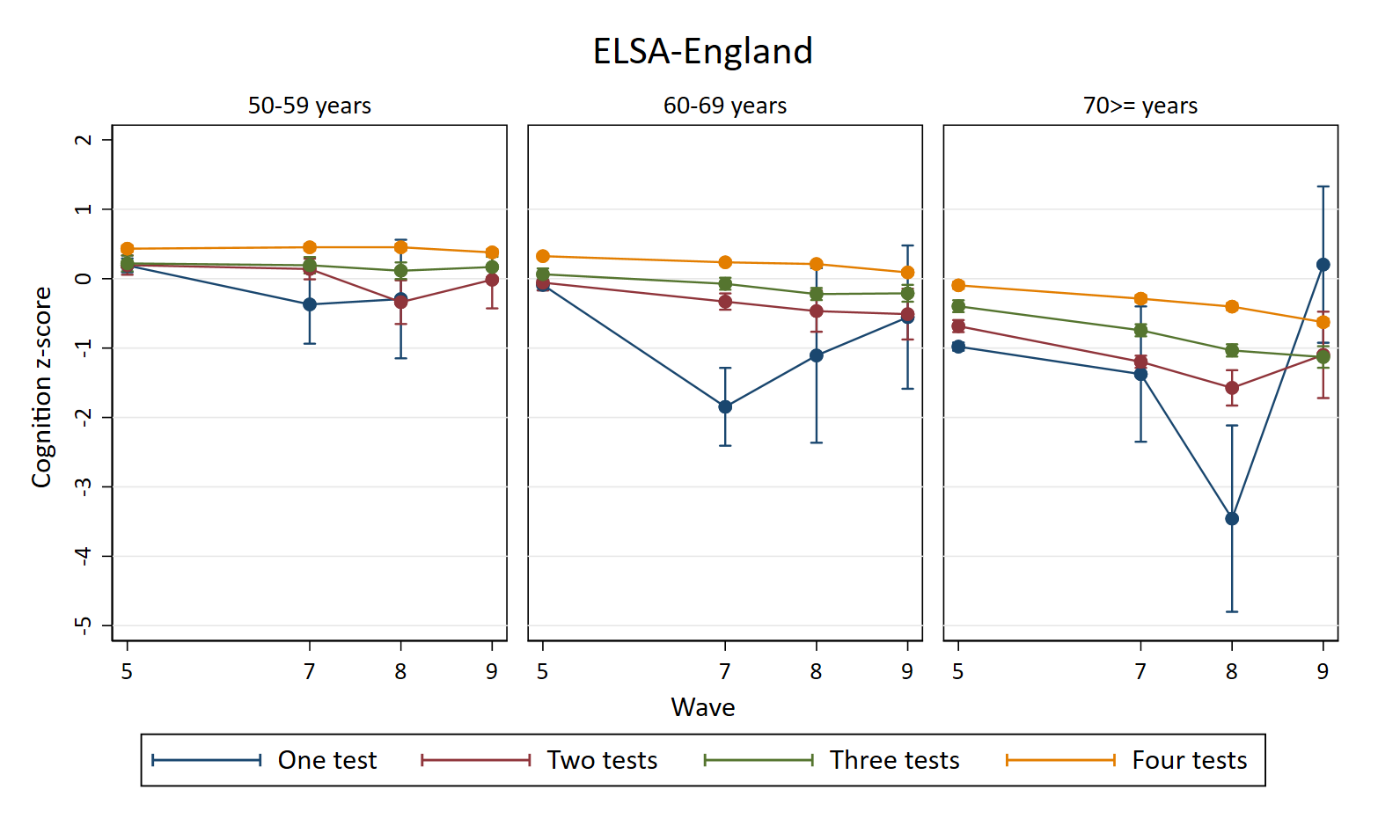


## Supplementary Figure 2 Predicted 7-year age trajectories in cognition z-scores by educational level according to country and gender (Model 3)


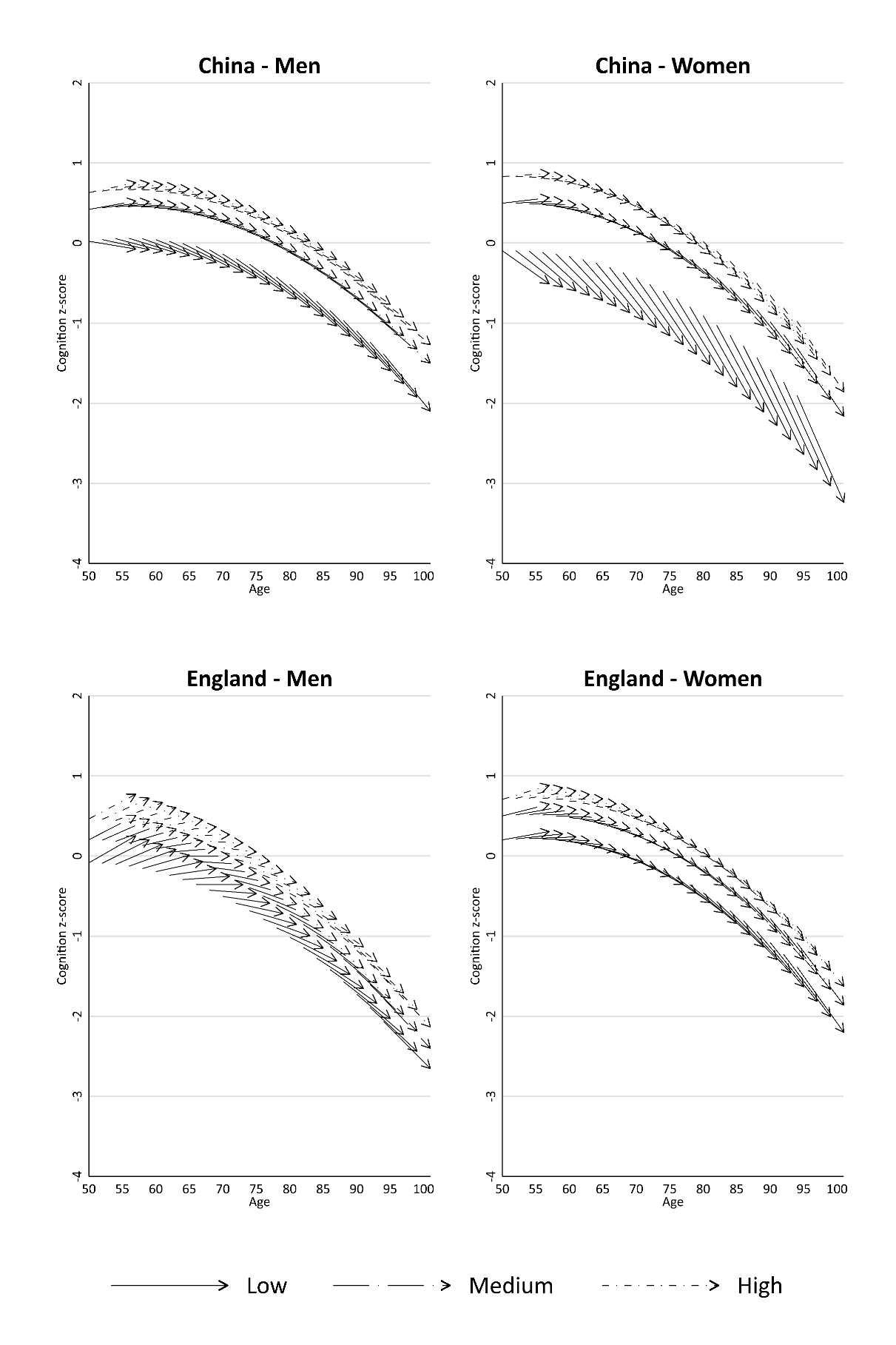


## Supplementary Figure 3 Predicted 7-year age trajectories in total cognition z-scores by educational level according to country and gender (Model 1)


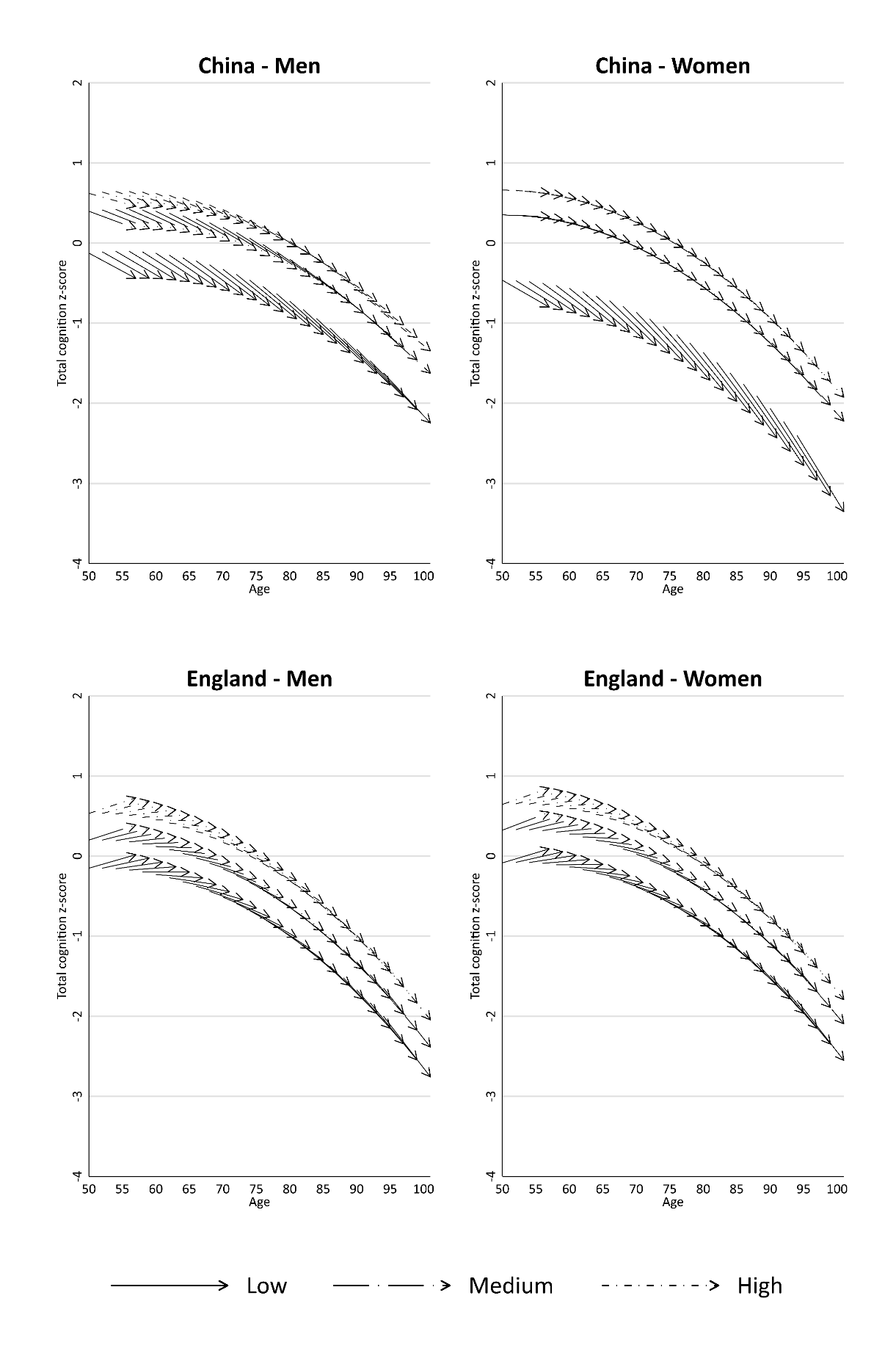


## Supplementary Figure 4 Predicted 7-year age trajectories in total cognition z-scores by educational level according to country and gender (Model 3)


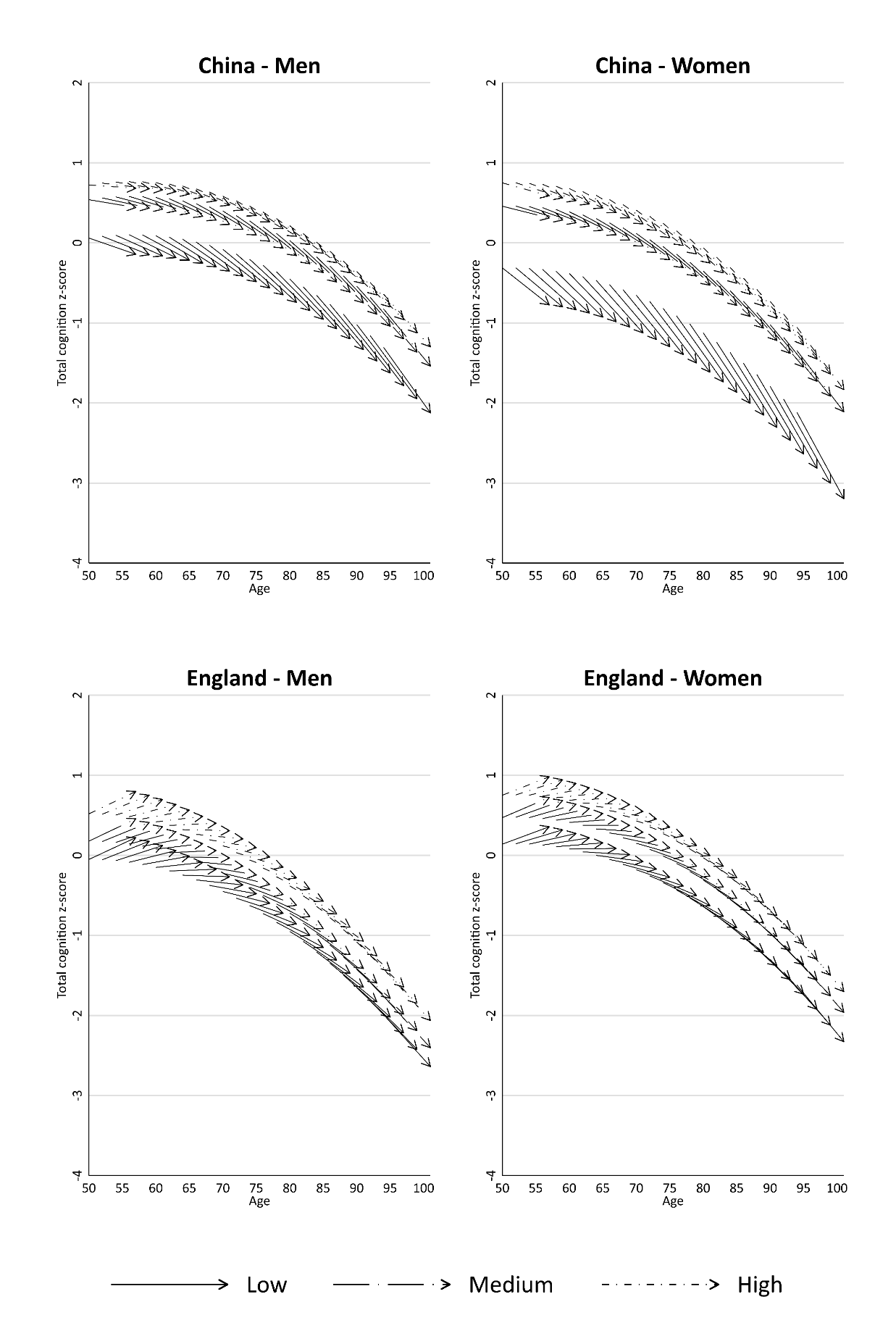

Supplement: igad064_suppl_Supplementary_Material [file igad064_suppl_supplementary_material.docx]
